# Imaging protoporphyrin IX photoproduct accumulation as a dosimetry reporter for monitoring photodynamic therapy of oral cancer

**DOI:** 10.1117/1.JBO.30.S3.S34114

**Published:** 2025-12-09

**Authors:** Christian Liboy, Shakir Khan, Bofan Song, Deshawn Vega, Mohammad A. Saad, Rongguang Liang, Tayyaba Hasan, Jonathan P. Celli

**Affiliations:** a Boston College, Boston, Massachusetts, United States; b University of Massachusetts Boston, Boston, Massachusetts, United States; c Massachusetts General Hospital, Harvard Medical School, Wellman Center for Photomedicine, Boston, Massachusetts, United States; d University of Arizona, Wyant College of Optical Sciences, Tucson, Arizona, United States; e Harvard University and Massachusetts Institute of Technology, Division of Health Sciences and Technology, Cambridge, Massachusetts, United States

**Keywords:** photodynamic therapy, 5-aminolevulinic acid (ALA), protoporphyrin IX photoproducts, photobleaching, oral cancer, photodynamic therapy dosimetry

## Abstract

**Significance:**

Photodynamic therapy (PDT) for the treatment of oral cancers and oral potentially malignant lesions can be enhanced by the capability of the photosensitizer to serve as a fluorescence contrast agent for treatment guidance. The development of image-based dosimetry reporters can inform treatment progress in real time to avoid under-treatment, leading to incomplete response and recurrence.

**Aim:**

We investigate the hypothesis that imaging of protoporphyrin IX (PpIX) photoproduct (PP) accumulation may be leveraged as an implicit PDT dosimetry reporter for PDT using 5-aminolevulinic acid (ALA)-induced PpIX photosensitization.

**Approach:**

In initial spectroscopy studies, we investigate dose-dependent changes in absorption and fluorescence spectra of PpIX corresponding to PP accumulation during red light (635 nm) delivery. We use spectral analysis to select fluorescence excitation and spectral filtering components for PP imaging during treatment. We evaluate the capability for imaging PP accumulation concomitant with PpIX photobleaching in tissue phantoms, 3D oral squamous cell carcinoma (OSCC) models, and in murine xenografts.

**Results:**

Spectroscopy shows fluence-dependent changes in PpIX optical properties, and that excitation of photobleached PpIX with 450 nm light produces fluorescence emission associated with PpIX PPs. An existing handheld intraoral probe is shown to be capable of imaging dose-dependent PP accumulation with the addition of a spectral filter to isolate fluorescence emission longer than 650 nm. PP signal increases concomitant with PpIX photobleaching in a fluence-dependent manner and correlates with the extent of cytotoxic response in 3D cultures. PP accumulation is also observed to occur concomitantly with photobleaching in OSCC subcutaneous xenografts.

**Conclusions:**

Overall, the results show that imaging of PP accumulation is feasible by adapting traditional photodiagnosis optical components and may serve as a dosimetry reporter for ALA-PDT, which is complementary to the measurement of PpIX photobleaching.

## 1 Introduction

In this study, we investigate a treatment monitoring approach for photodynamic therapy (PDT) of oral lesions based on fluorescence imaging of the accumulation of protoporphyrin IX (PpIX) photoproduct (PP) during light delivery. This is motivated by the need for new treatment options for oral squamous cell carcinoma (OSCC), which has persistently high incidence and mortality rates, with more than 389,000 cases per year and 188,000 deaths annually according to the most recent GLOBOCAN estimates.^1^ The premalignant lesions (oral potentially malignant disorders, OPMD) from which OSCC arises are ubiquitous, with an overall worldwide prevalence of 4.47%.^2^ Even when detected early, clinical management of these oral lesions is confounded by the high uncertainty of malignant transformation. Surgical excision comes with potential morbidity that may not be justified, but the alternative to “watch and wait” comes with the risk of a missed opportunity to treat. The situation is particularly dire in South Asia. India alone accounts for about one-third of the global incidence of OSCC, where the impact is exacerbated by inadequate access to oral surgery and radiation oncology for millions of patients.^3^ For all of these reasons, there is urgency to address the unmet need for an effective minimally invasive intervention for oral lesions, which can be implemented with reliable dosimetry tools that facilitate treatment success without extensive treatment planning that is not feasible in resource-limited settings.

Photodynamic therapy (PDT) is a light-based treatment modality in which target tissue is photosensitized, then illuminated with an appropriate wavelength to initiate photochemical reactions leading to lethal accumulation of reactive molecular species at the lesion site.^4^ PDT has been shown to be clinically effective for both OPMD and early-stage oral cancers,^5^^–^^7^ though as yet no standardized protocols exist for PDT of oral lesions. The ongoing development of technologies that streamline intraoral light delivery and leverage the established utility of photosensitizer fluorescence for treatment guidance and monitoring holds promise to pave the way for the broader adoption of PDT for oral lesions.^4^

It is with this motivation that we have investigated and reported on fluorescence imaging tools to enable image-guided intraoral PDT for use with 5-aminolevulinic acid (ALA)-induced PpIX photosensitization.^8^^–^^10^ The integration of simultaneous fluorescence imaging of the illuminated field during PDT not only facilitates guidance of light delivery but also leverages the ability of photosensitizer photobleaching to serve as a dosimetry surrogate.^11^ The expectation is that photobleaching occurs in proportion to the amount of reactive molecular species (RMS) generated and therefore in proportion to the cytotoxic dose deposited. This approach has been demonstrated with some notable successes. For example, in a human trial using interstitial PDT for malignant glioma, Johansson et al.^12^ showed that the extent of PpIX photobleaching correlated directly with outcome. Our team reported a similarly strong correlation (p<0.001) between the extent of PpIX photobleaching and tumor response in early-stage oral cancers.^6^ Detailed investigation of photobleaching as a corollary of dose deposited has shown, however, that it is often difficult to extrapolate a reproducible relationship between the photobleaching and dose response because there is often a dramatic drop in fluorescence over a narrow dose range.^10^^,^^13^ This is especially true for highly photo-unstable compounds such as PpIX. The quantification of a decreased fluorescence signal is also confounded by the many other factors that can cause varying attenuation of photosensitizer fluorescence intensity at different time points in a sequence of images.

The potential for photobleaching as a high-precision dosimetry reporter may be enhanced by correlating it with other changes in optical properties, which also occur in a dose-dependent manner. In the case of protoporphyrin IX, photooxidation reactions involving PpIX itself are known to lead to the formation of various photoproducts (PP), which correspond to the emergence of new absorption and fluorescence emission peaks.^14^^–^^17^ The prominent chlorin-like photoproduct of PpIX exhibits an absorption peak at around 673 nm, which is not present in PpIX prior to light exposure, and also exhibits increased fluorescence at deeper red wavelengths.^17^ More recently, Ogbonna et al.^18^ showed that this photoswitching may be leveraged in image-guided surgery, providing deeper tissue fluorescence than PpIX prior to light exposure.

Here, we investigate the potential of imaging PP accumulation during light delivery as a dosimetry reporter for intraoral PDT. The central hypothesis is that using appropriate optics to separate the fluorescence signals associated with PpIX and PP, it should be possible to observe an increasing PP signal concomitant with PpIX photobleaching as part of a more complete optical signature of the light dose deposited than either signal alone. This proof-of-principle study adopts our existing oral probe for PDT light delivery, combined with components needed for simultaneous imaging of fluorescence from PpIX and its photoproduct. We present data evaluating this approach in tissue phantoms, 3D cell culture models of OSCC, and in pilot measurements in a small number of murine tumor xenografts. In the latter, we adopt a subcutaneous tumor skin flap model, which allows fluorescence imaging of the tumor tissue with the skin pulled back during treatment. This model enables the skin to be sutured post-treatment, allowing for the correlation of tumor response over a 1-week follow-up period with the fluorescence signature reported as a prognostic indicator at the time of treatment.

## 2 Materials and Methods

### 2.1 UV–Visible Spectroscopy Studies

Absorption spectra for PpIX were obtained using a VWR PV4 spectrophotometer (VWR International, Radnor, Pennsylvania, United States). All measurements were carried out using purified PpIX (P8293, Sigma-Aldrich Inc., St Louis, Missouri, United States) dissolved in DMSO (276855, Sigma-Aldrich Inc., St Louis, Missouri, United States), and readings were performed on PpIX solutions in a quartz cuvette. For analysis of spectroscopic changes due to light exposure, PpIX solutions, initially prepared under reduced room lighting in 35 mm petri dishes, were exposed to a specified fluence of red light (635 nm) using a previously reported and clinically validated fiber-coupled light-emitting diode (LED) light source designed for intraoral PDT,^19^ to mimic photobleaching under clinical conditions. Light was delivered to samples placed on a stage above a vertically mounted fiber optic with a collimating lens to achieve a uniform beam spot slightly overfilling the petri dish with an irradiance of $60\;\mathrm{mW}/{\mathrm{cm}}^{2}$. For power calibration, measurements were performed using a Thorlabs power/energy meter (PM100D, Thorlabs, Newton, New Jersey, United States) with a standard silicon photodiode power sensor (Thorlabs, S121C). Calculation of quoted irradiance was obtained from raw power readings based on the 9.5 mm diameter circular sensor (area of $0.709\;{\mathrm{cm}}^{2}$). The UV–Vis spectra were measured in 1.0 nm intervals in the range of 330 to 800 nm.

### 2.2 Fluorescence Spectroscopy Studies

Fluorescence emission spectra were obtained using a JASCO FP-8350 spectrofluorometer (JASCO Inc., Easton, Massachusetts, United States) in the Biophysical Instrumentation Core (BIC) at the University of Massachusetts Boston. To compare PDT dose-dependent changes in fluorescence spectra, PpIX solutions (in DMSO solvent) were prepared in petri dishes and exposed to 635 nm light under controlled conditions using the same light delivery protocol as described above. A 1% solution of Triton-X 100 (TX1568-1, Sigma-Aldrich Inc., Allentown, Pennsylvania, United States) was used for lysis of post-PDT 3D culture tissues for PpIX/photoproduct fluorescence emission spectra. The fluorescence spectroscopic parameters were set with excitation wavelength 450 nm and reading range 460 to 750 nm and for 405 nm excitation reading range was set to 420 to 750 nm.

### 2.3 Intraoral Imaging Devices

For imaging of PpIX fluorescence and photobleaching, we used a previously reported smartphone-controlled intraoral imaging device, which was originally developed for oral cancer screening in low-resource settings.^20^^,^^21^ Briefly, the imaging head has a pair of 5-megapixel OV5648 Complementary Metal-Oxide-Semiconductor (CMOS) sensors (OV5648, OmniVision Technologies, Santa Clara, California, United States), as shown in Fig. 3, to allow both pWLI (polarized WL) and fluorescence imaging without moving parts. The 405 nm LED provides excitation for autofluorescence imaging and PpIX fluorescence (exposure time of 30 ms with irradiance of $22\;{\mathrm{mW}/\mathrm{cm}}^{2}$ and total fluence of $0.6\;{\mathrm{mJ}/\mathrm{cm}}^{2}$). For fluorescence imaging, a short-pass 425 nm filter (Asahi Spectra, Tokyo, Japan) is mounted in front of the 405 nm LED, whereas a wide band-pass 465 to 650 nm filter (Asahi Spectra, Tokyo, Japan) is mounted over the camera. This allows imaging of autofluorescence and PpIX signal in the green and red channels, respectively, of the OV5648 as reported previously.^8^ The white LED provides illumination for polarized white-light images, which provide detailed surface information of the oral tissue, as in our original diagnostic probe, which is further improved by placing the orthogonal linear polarizers (Edmund Optics, Barrington, New Jersey, United States) in front of each white light LED.

For imaging of PpIX photoproduct (PP), an additional, modified version of the device was used. This probe had a 650 nm long-pass filter over one of the cameras. As reported by Ogbonna et al. ^18^ and also shown in the spectroscopic studies presented herein, 450 nm is used for preferential excitation of PP in tissues containing photobleached PpIX. Since the existing handheld intraoral probe does not provide 450 nm excitation, a Thorlabs M450LP1 LED source (Thorlabs) was mounted above the sample to provide 450 nm excitation (exposure time of 2 s with irradiance of $2.9\;{\mathrm{mW}/\mathrm{cm}}^{2}$ and fluence of $5.8\;{\mathrm{mJ}/\mathrm{cm}}^{2}$).

Images were obtained using a standard lab ring stand/clamp to hold each imaging device, taken at a fixed distance (10 mm) above the sample in question, prepared in either 96 well black walled plates (655090, CELLSTAR® μCLEAR® 96-Well Polystyrene Tissue Culture Treated Multiple Well Plates, black walled, clear bottom, Greiner Bio-One, Frickenhausen, Germany) or 35 mm dishes (150318, Thermo Fisher BioLite Cell Culture Treated Dishes, Roskilde, Denmark) or 35 mm glass bottom dishes with 10 mm diameter cover slip bottom inset (P35G-1.5-10-C, MatTek corporation, Ashland, Massachusetts, United States). Fluorescence image analysis was performed using open-access FIJI ImageJ software. The intraoral device generates images with 1344×1792  pixel dimensions and an 8-bit depth RGB. The raw RGB images were split into separate channels for further analysis.

### 2.4 Preparation of Tissue Phantoms Containing PpIX

Solid agarose-based phantoms were prepared by mixing 0.5 g/l ${\mathrm{TiO}}_{2}$ (Titanium(IV) Oxide, 277370010, Acros Organics/Thermo Fisher Scientific, Haverhill, Massachusetts, United States) into 40°C molten 1% agarose (0710, VWR Chemicals, Solon, Ohio, United States) as a tissue scattering mimic mixed with PpIX at room temperature in PBS. A ${\mathrm{TiO}}_{2}$ concentration of 0.5 g/l was selected to achieve a scattering coefficient, $\mu_{s}$, of $9.1\;{\mathrm{cm}}^{-1}$ for 630 nm light, based on the literature,^22^^,^^23^ and consistent with tissue optical property measurements of oral lesions.^24^ The concentration of PpIX used was 40  μM, which is within the range of reported tumor tissue PpIX concentrations (0.2 to 50  μM) after ALA administration.^25^ Then, when the temperature reduced to 35°C, 1.0 g/dl hemoglobin (Bovine hemoglobin, H2625, Sigma-Aldrich Inc., St Louis, Missouri, United States) was added and homogeneously mixed with PpIX in the presence of 0.05% Tween-20 (P1379, Sigma Aldrich Inc., Milwaukee, Milwaukee, United States).^26^^,^^27^ Here, the incorporation of 0.1 g/dl Hb is used to recapitulate tissue-like absorption coefficient of $∼1.2\;{\mathrm{cm}}^{-1}$ at 630 nm based on the literature.^22^^,^^24^^,^^28^ Although still in the liquid phase, the agarose mixture was cast into a 35 mm dish for solidification prior to use in imaging experiments. Experiments involving light delivery to phantoms used the same 635 nm (peak) LED source to illuminate samples with irradiance of $60\;\mathrm{mW}/{\mathrm{cm}}^{2}$ as described for the spectroscopy studies above.

### 2.5 OSCC Tissue Culture Models

3D cell cultures embedded in tissue-simulating medium were prepared using TR146 cells (Cat. no. ECACC 10032305, purchased from Sigma-Aldrich), an established OSCC cell line derived from a neck metastasis of a buccal mucosa tumor, following a protocol reported previously.^10^ TR146 stocks were maintained and subcultured in T75 flasks maintained at 37°C, 5% ${\mathrm{CO}}_{2}$, and 95% humidity in Ham’s F-12K (Kaighn’s) medium containing l-glutamine (10-080-CV, Corning, Corning, New York, United States) supplemented with 1% Penicillin/Streptomycin (15140-122, Gibco™, Thermo Fisher Scientific, Waltham, MA, USA), 250 ug/ml amphotericin B (30-003-CF, Corning®, Bedford, Massachusetts, United States) and 10% Fetal Bovine Serum (A52568-01, Gibco, Life Technologies Corp., Grand Island, NY, United States). To generate 3D models, TR146 cells were trypsinized with 0.25% Trypsin protease (SH30042.01, Cytiva, HyClone, Logan, Utah, United States), harvested, resuspended in DPBS (Dulbecco's Phosphate Buffer Saline, SH30028.02, Cytiva, HyClone, Logan, Utah, United States), and pelleted by centrifugation before being embedded in alginate hydrogels containing supplements to better mimic the optical properties of tumor tissue. In this protocol, 3 mM ALA (A7793, Sigma-Aldrich Inc., St. Louis, Missouri, United States) was delivered to cells in culture 4 h prior to trypsinization so that there would be sufficient PpIX accumulation by the time of imaging. After harvesting the cells in DPBS (${\mathrm{Ca}}^{2+}$, ${\mathrm{Mg}}^{2+}$ free), 6 to $10\times 10^{6}$ cells were formed into a pellet after 5 min centrifugation at 500×g for mixing into 0.1% (w/v) sodium alginate (180947, Sigma-Aldrich Inc., Milwaukee, Milwaukee, United States). The alginate mixtures were crosslinked by 100 mM ${\mathrm{CaCl}}_{2}$ in DPBS (${\mathrm{Ca}}^{2+}$, ${\mathrm{Mg}}^{2+}$ free) solvent as described in earlier studies.^29^^–^^31^ In some samples, the changes in optical properties of endogenous PpIX were confirmed by spectroscopy analysis of extracted PpIX. In these samples, alginate-embedded TR146 cell aggregates were dissolved in 0.05 M sodium citrate solvent (567446, Sigma-Aldrich Inc., St. Louis, Missouri, United States), and cells were pelleted (300×g) and suspended in DPBS (${\mathrm{Ca}}^{2+}/{\mathrm{Mg}}^{2+}$ free). For viability analysis, 40 k cells were taken from the suspension. For fluorescence spectral analysis, the same suspended cells were again pelleted and suspended in 1% Triton X-100 to extract PpIX and photoproducts following a protocol previously described.^32^

*In vitro* PDT experiments used the same LED source and calibration protocol as described for the spectroscopy studies above. For correlation of fluorescence signals and cytotoxic response to PDT, the separated 40,000 cells re-plated into 96-well plates, incubated for 24 h for quantitative viability analysis, where fluorescent vital dyes calcein AM (Cell-permeant Green, C3100MP, Invitrogen, Carlsbad, California, United States) and ethidium bromide (Ethidium Bromide Solution, H5041, Promega Corporation, Madison, Wisconsin, United States) were used as an terminal endpoint for quantification of cytotoxic response, based on a methodology previously reported.^33^

### 2.6 Murine Xenograft Experiments

Athymic nude mice (strain: 007850, homozygous Foxn1), aged 6 to 8 weeks and weighing 20 to 25 g, were obtained from the Jackson Laboratory (Bar Harbor, Maine, United States). The animal study protocol was approved by the Institutional Animal Care and Use Committee (IACUC) at UMass Boston, according to the guidelines established by the NIH. Prior to implantation, the cells were cultured in a 150 mm cell culture dishes (130183, BioLite, Thermo Scientific, Rochester, NY, United States) at 37°C in Ham’s F-12K medium, then trypsinized and harvested when cell density reached around $20\times 10^{6}$ cells/ml. Mice were anesthetized using isoflurane inhalation during the subcutaneous implantation. The right flank of each mouse was injected with $6\times 10^{6}$ cells in a 50  μl volume containing a 1:1 ratio of culture media and Growth Factor Reduced Matrigel Basement Membrane Matrix (356231, Matrigel Matrix, Corning Life Sciences, Bedford, MA, United States). Implanted tumors were allowed to grow until they reached 5 to 7 mm in diameter (∼65 to $180\;{\mathrm{mm}}^{3}$ in volume) at 12 days after implantation. The tumor size was measured by vernier caliper using the ellipsoid volume calculation formula (V=π/6·l·w·h). On day 12, ALA (200 mg/kg) was injected intratumorally (i.t.) 2.5 h before the light delivery procedure. Prior to PDT treatment and monitoring, mice were anesthetized by isoflurane inhalation. Anesthesia was initially induced with 2.5% isoflurane and maintained at 1.5% isoflurane mixed with oxygen (Medical grade $O_{2}$ cylinder, Airgas) under an oxygen flow rate of 1.8 l/min at 14.7 psi. To enable fluorescence imaging, a small arc-shaped incision (6- to 10-mm) was made adjacent to the tumor to allow opening of a skin flap and exposure of the tumor tissue by applying a Guthrie skin hook retractor. All PDT-treated mice received a fluence of $100{J/\mathrm{cm}}^{2}$ at the tissue surface (an irradiance of $60\;{\mathrm{mW}/\mathrm{cm}}^{2}$ over a spot of 1 cm diameter delivered from the same 635 nm fiber-coupled LED light source as described above, where the fiber with collimator lens was mounted vertically above the mouse). Power measurements were performed as described above for *in vitro* studies; in this study, the silicon photodiode sensor was placed as nearly as possible at the same height as the mouse tumor tissue surface. Fluorescence imaging was performed at the tumor site immediately before and after light delivery, before closing and suturing the skin flap. Drops of saline were used to keep the exposed tissue moist during procedures, and 6-0 sutures were used to close the flap. In addition, a transparent adhesive film dressing (Tegaderm, 3M Health Care Company, St. Paul, Minnesota, United States) was used to cover the incision area during its healing process. After treatment, mice were allowed to recover, and ongoing caliper measurements were performed to monitor tumor volume post-treatment. The tumor size at each time point was measured by vernier caliper using the ellipsoid volume calculation formula (V=π/6·l·w·h).

### 2.7 Statistical Analysis

R packages (Comprehensive R Archive Network) were used to calculate the difference in central values of the resulting data. After image segmentation using open-source Fiji ImageJ,^34^ input parameters were analyzed, including PpIX fluorescence, photobleaching, and photoproduct fluorescence, to investigate correlations between PP accumulation, photobleaching, dose delivered, and dose response in each model system. The statistical significance of differences between mean values was analyzed using the Kruskal–Wallis test, and a Dunn post hoc analysis was performed at a Bonferroni-adjusted p-value of 0.05. The correlation matrix was calculated using the Python Pandas package’s corr() method and visualized using the Seaborn library. Significance levels were set according to convention; p-values: ***p<0.001; **p<0.01; *p<0.05. To analyze correlations between fluorescence signals and cytotoxic responses to PDT, regression plots were created using Python’s Seaborn package on top of Matplotlib.pyplot module. The regression $R^{2}$ (confidence interval or CI = 95%, shown as shaded area in the plots) and p-value were calculated using the scipy package’s “stats” method applied to the dataset of PP spectral signals and PP fluorescence values of images.

## 3 Results and Discussion

### 3.1 Characterization of Fluence-Dependent Changes in PpIX Optical Properties

**Fig. 1:**
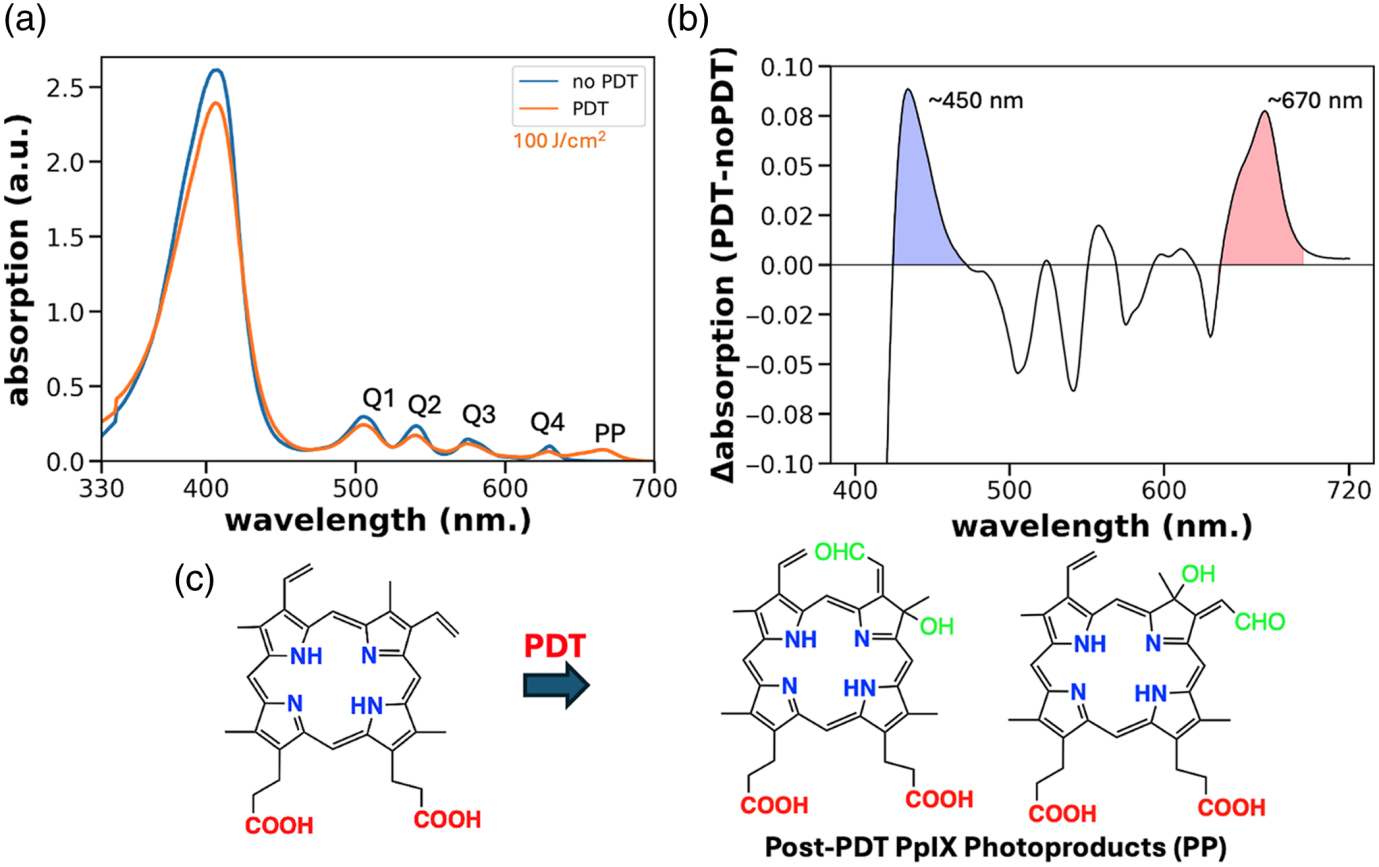
Comparison of representative absorption spectra for samples of PpIX in DMSO maintained in dark conditions and photobleached under 635 nm LED light delivery ($100\;J/{\mathrm{cm}}^{2}$). In panel (a), the UV–visible absorption spectra (350 to 700 nm) are overlaid. The subtraction spectrum in panel (b) shows the emergence of new absorption peaks at 450 and 673 nm, consistent with previous characterization of PpIX photoproducts reported in Ref. 15 (c).

We performed UV–Vis and fluorescence spectroscopy studies of PDT in solvents to evaluate PP accumulation under clinically relevant light exposure conditions for PDT treatment of oral lesions (fluence of 0 or $100\;J/{\mathrm{cm}}^{2}$, delivered at irradiance of $60\;\mathrm{mW}/{\mathrm{cm}}^{2}$) (Fig. 1). This analysis was repeated in four independent runs with the same light delivery parameters (see Figs. S1 and S3 in the Supplementary Material) Spectra were obtained for PpIX solutions exposed to light delivered from a 635 nm LED source previously reported for intraoral PDT.^19^ Subtraction of the baseline (dark) PpIX spectra from photobleached PpIX shows emergence of peaks at 450 and 673 nm, consistent with accumulation of chlorin-like photoproducts as previously reported.^17^ Chemical structures shown in Fig. 1(c) are based on characterization of PpIX photoproducts reported by Ogbonna et al.^15^ Additional UV–Vis spectroscopy data for PpIX in DMSO and methanol at varying fluence of 635 nm light are shown in Fig. S2 in the Supplementary Material.

**Fig. 2:**
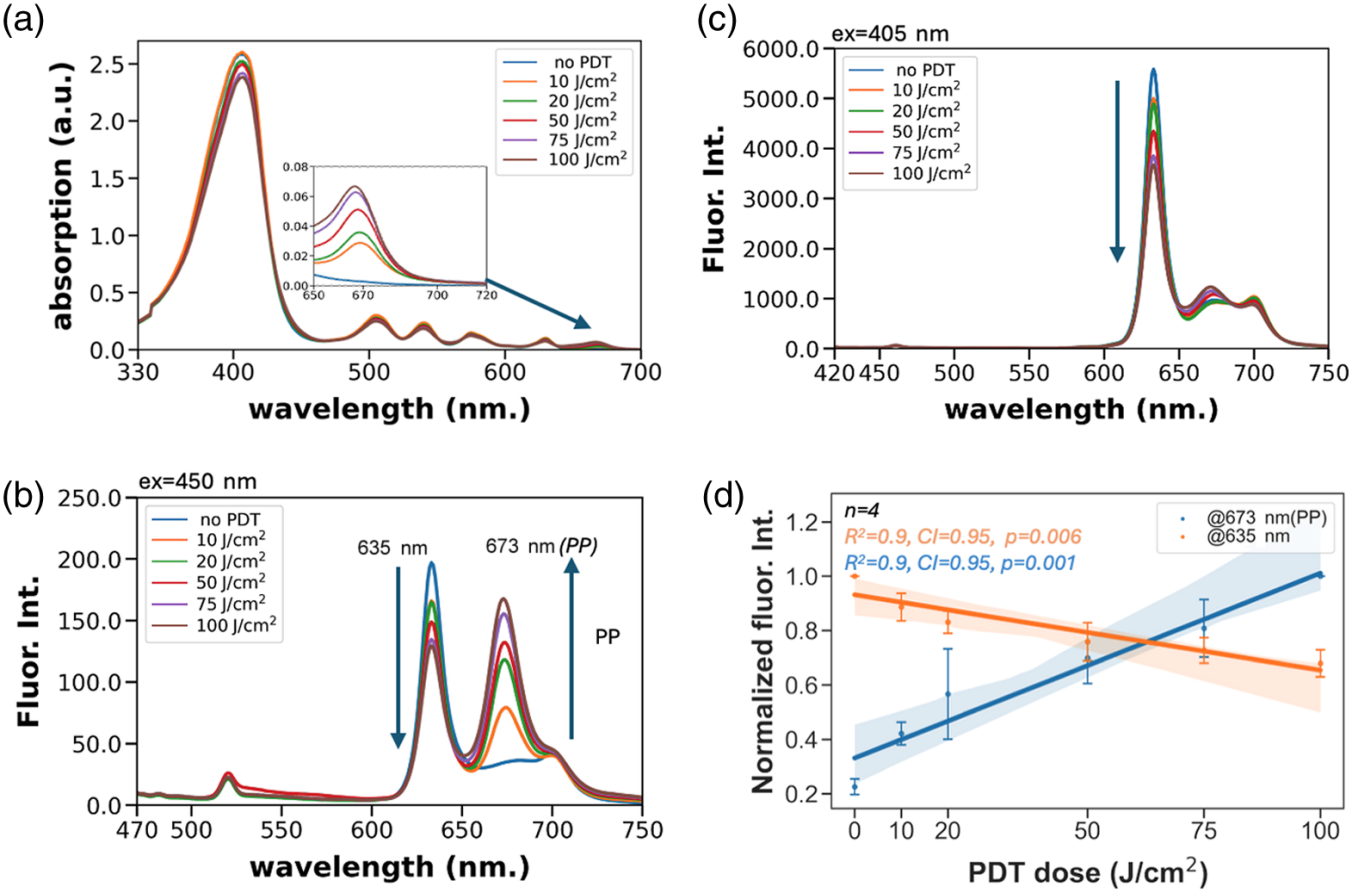
Spectroscopy analysis of PpIX samples exposed to varying fluence from a 635 nm LED light source. (a) Absorption spectra for PpIX in DMSO exposed to varying fluence, showing a dose-dependent increase in absorbance at 673 nm. (b) Excitation with 405 nm light (PpIX Soret band) shows characteristic dose-dependent photobleaching. There is also a systematic shift in the characteristic dual-peaked PpIX emission pattern, with a decrease in emission below 650 nm but a slight increase in the longer-wavelength emission (673 nm) peak. (c) Excitation at 450 nm produces lower overall emission intensity but shows a dramatic increase in long wavelength fluorescence above 650 nm presumably due to a higher proportion of activation of PpIX photoproducts (PP). The dose-dependent amplitude of the two emission peaks averaged over four experiments is shown in panel (d).

Furthermore, the same samples, after UV–Vis reading, were analyzed using fluorescence spectroscopy. The samples were diluted to 0.5  μM in DMSO, applying the reading parameters as stated in Sec. 2. Fluorescence spectroscopy (Fig. 2) using excitation at 405 nm (a traditional choice for excitation of PpIX at the Soret band) shows a systematic decrease in fluorescence consistent with photobleaching. Additional spectra were obtained with 450 nm excitation to preferentially activate photoproduct fluorescence. Here, a decisive shift in the characteristic dual-peaked red emission of PpIX is observed, with the peak at around 635 nm decreasing, whereas the peak at 670 to 675 nm increases in a consistent dose-dependent manner, as shown in Fig. 2(d). This same trend is present, though less obviously visible, with 405 nm excitation as well. This is consistent with the picture that the photobleached PpIX is actually a mixture of multiple compounds with differing optical properties and differing contributions to fluorescence emission depending on the wavelength of excitation. Overall, the systematic dose-dependent changes in optical properties under these controlled conditions support the feasibility of using 450 nm excitation for PP accumulation, combined with spectral filtering to isolate longer-wavelength emission, as an optical reporter of the dose deposited. The data shown are averaged from four independent experimental runs with the same PDT and spectral reading parameters (see Fig. S3 in the Supplementary Material).

To visualize both PpIX photobleaching and concomitant increase in fluorescence signal from the formation of PpIX photoproducts, an adaptation of previously reported smartphone-controlled intraoral imaging devices was used. An existing intraoral probe was used for excitation of PpIX fluorescence using an embedded 405 nm LED in the probe tip and imaging of red fluorescence emission on the CMOS camera. A clone of that device was prepared, with the emission filter over the CMOS camera replaced with a 650 nm long pass filter to isolate the longer wavelength PP-associated emission. The second device, lacking an embedded 450 nm source, was used in conjunction with an external 450 nm LED mounted above. All samples were treated using a previously clinically validated 635 nm fiber-coupled LED light source, as shown to model therapeutic light delivery in phantoms, tissues, and mice throughout this study.

### 3.2 Imaging PpIX Photoproduct Accumulation in Tissue Phantoms Using Intraoral Probe

**Fig. 3:**
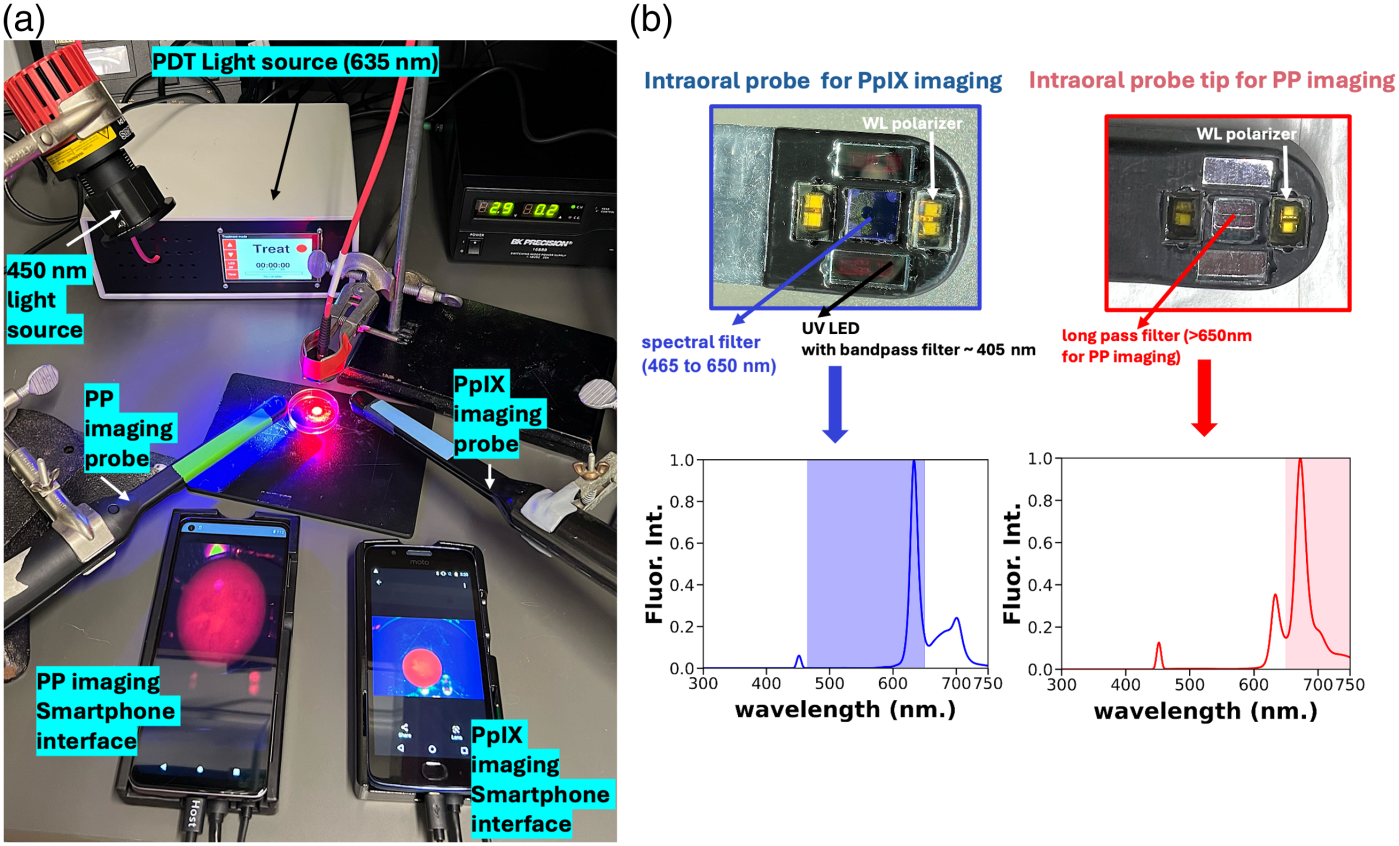
Imaging setup to visualize PpIX and PP fluorescence emission using intraoral imaging and PDT hardware. (a) Action image of OSCC tissue model ALA-PDT and PpIX and post PDT photoproduction (PP) and photobleached imaging. The connected smartphone app is used to monitor the treatment and PDT guidance. (b) The fluorescence imaging probes attached to smartphones show the LED light source components and bandpass filters corresponding to the tumor site produced and bleached PpIX, as well as photoproduct (pre- and post-PDT) correlated to their fluorescence spectral range.

To evaluate image guidance based on PP accumulation, we initially performed tests using optical phantoms and obtained images using an adaptation of our existing smartphone-controlled hardware (Fig. 3). PDT light delivery was performed using the fiber-coupled light source of Liu et al,^19^ whereas images were obtained with the oral probe mounted above the phantom. Unless otherwise noted, the beam spot was calibrated to an irradiance of $60\;\mathrm{mW}/{\mathrm{cm}}^{2}$, and the duration of light delivery was calculated for the indicated fluence.

As the existing hardware was built for PpIX excitation at 405 nm, an external 450 nm LED illuminator was mounted above for PP activation based on the spectroscopy analysis above. The existing bandpass filter for PpIX emission was swapped with a 650 nm long-pass filter. However, as it was also desirable to compare side by side with the traditional PpIX imaging intraoral device, we also mounted a clone of the existing oral camera (with 405 nm excitation) alongside the modified one.

**Fig. 4:**
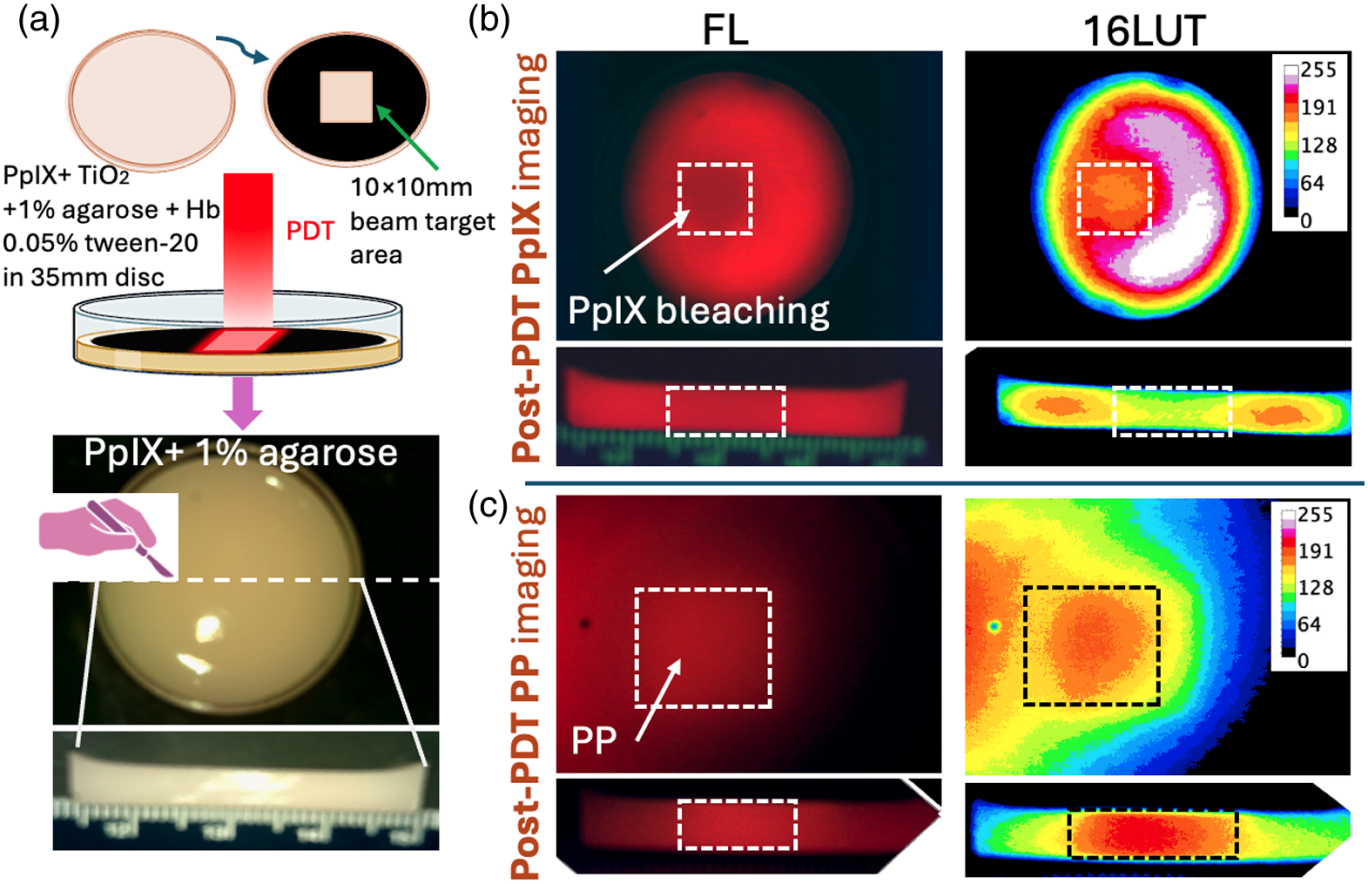
Imaging PpIX photobleaching and photoproduct formation in optical phantoms. (a) To visualize spatial patterns of photobleaching and photoproduct accumulation, solid agar phantoms containing hemoglobin in addition to PpIX and ${\mathrm{TiO}}_{2}$ were treated with 635 nm light delivery to model clinical PDT. Here, the solid agar was also cut cross-sectionally after light exposure to examine depth-dependent fluorescence profiles. Graphic created in BioRender.^35^ (b) and (c) Image panels show the multichannel CMOS camera images of solid agar phantoms imaged with the PpIX and PP filters (FL) alongside the red channel alone, displayed using a 16-color lookup table (abbreviated 16LUT) to visualize contrasting surface photobleaching and PP signatures. The representative phantom shown was treated with a fluence of $100\;J/{\mathrm{cm}}^{2}$. The lower panels show the corresponding cross-sectional cuts of each phantom. The photoproduct formation is clearly evident in the cross-sectional cut view.

Using this approach, we co-registered dose-dependent changes in fluorescence signals attributed to photobleaching and PP formation from PpIX-containing solid agar optical phantoms with hemoglobin and ${\mathrm{TiO}}_{2}$ added to promote tissue-like absorption and scattering (Fig. 4). In these experiments, agar phantoms were also cut to expose the cross-sectional profile showing contrasting intensity profiles between the PpIX and PP imaging devices in the illuminated area. This is clearly visible in the contrast-enhanced (16 color lookup table/16LUT) display. The representative phantom shown in Fig. 4 received the highest fluence tested of $100\;{J/\mathrm{cm}}^{2}$. Additional phantoms (replicates; n=3) were treated with varying fluences of 635 nm light, and fluorescence signals were quantified and plotted against fluence, as shown in Fig. S4 in the Supplementary Material. This figure demonstrates that the signals associated with PpIX and PP decrease and increase, respectively, in a dose-dependent manner.

### 3.3 PpIX Photoproduct Accumulation Correlated with Treatment Outcome in 3D Cell Cultures

**Fig. 5:**
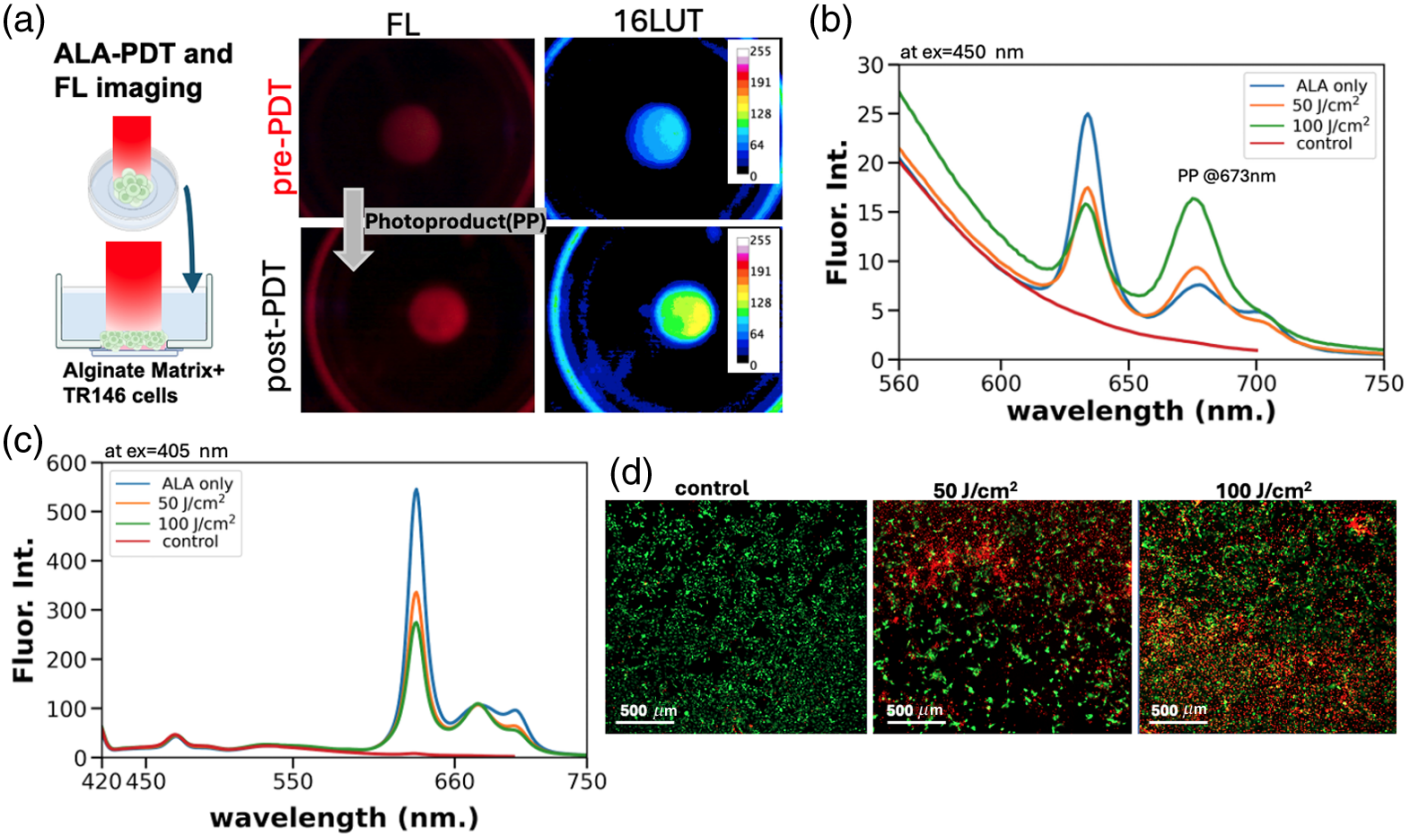
Imaging and spectral analysis of photoproduct accumulation following light delivery to ALA-photosensitized OSCC 3D cultures with endogenously produced PpIX. (a) Pre- and post-light delivery images for the raw fluorescence and contrast-enhanced displays of the PpIX channel are shown for representative TR146 3D cultures imaged using the modified oral imaging device for PP fluorescence emission. The pattern is consistent with what is shown above for the PpIX phantom. Graphic created in BioRender.^36^ (b) Fluorescence spectroscopy analysis of lysed tissues excited with 450 nm confirms an increase in longer wavelength emission (above 650 nm) in a dose-dependent manner, consistent with measurements on PpIX samples exposed to specified fluence above. (c) Fluorescence spectra of lysed tissue with 405 nm excitation are similar to what is seen in experiments on light-exposed PpIX in solvent. (d) Representative images of 3D cell cultures that were disaggregated and then replated for staining with vital dyes calcein AM and ethidium bromide (EtBr) to label live and dead cells (displayed in false color, green and red, respectively) and correlate later cytotoxic response with fluorescence signals measured at the time of treatment.

To assess the hypothesis that photoproduct accumulation is indicative of the dose deposited and eventual treatment outcome, we obtained PP fluorescence images from endogenously produced PpIX in live cells and correlated them with the PDT treatment response. These measurements used a protocol for embedding TR146 in an alginate scaffold supplemented with optical phantom components to promote tissue-like scattering and absorption in a 35 mm glass-bottom dish.^10^ Cultures were again treated using the fiber-coupled 635 nm light source. As shown in Figs. 5(a) and 5(b), the PP fluorescence signal increases after PDT. The control TR146 cells with no ALA and PDT show negligible PP fluorescence [see Fig. S5 in the Supplementary Material for fluorescence image]. Additional fluorescence spectroscopy analysis of lysed tissues is shown in Figs. 5(b) and 5(c) (see also Fig. S6 in the Supplementary Material). Imaging measurements performed using the intraoral device with a bandpass filter suitable for PpIX fluorescence showed strong photobleaching (Fig. S7 in the Supplementary Material), again supporting the expected correlation between photobleaching and PP accumulation.

**Fig. 6:**
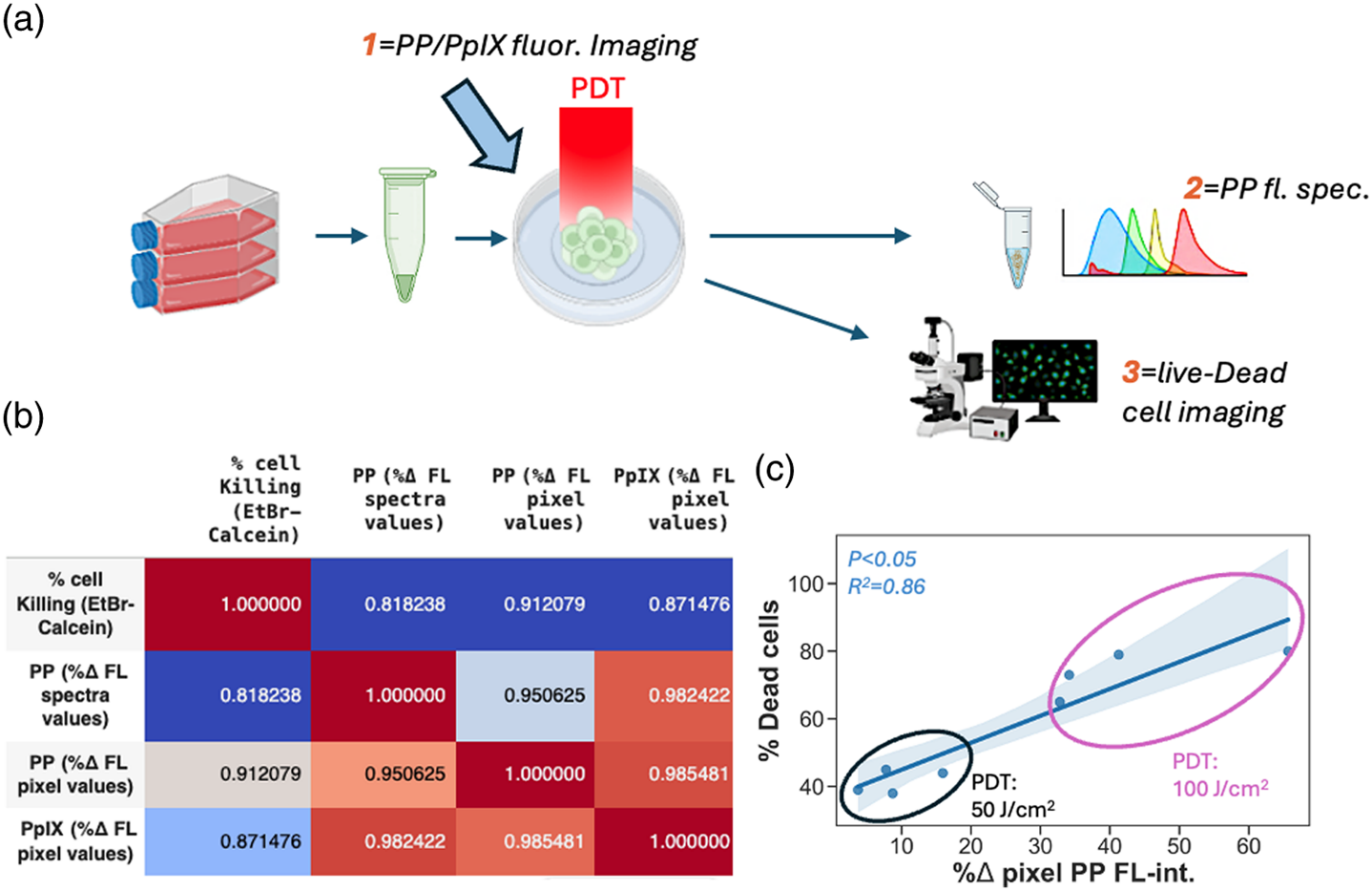
Correlation of PDT response with photoproduct accumulation in 3D cultures. (a) Schematic showing design of measurements to analyze the strength of correlation between potentially prognostic PP fluorescence signal at the time of treatment and dose response as measured by terminal staining of treated cells with vital dyes calcein AM and ethidium bromide. Graphic created in BioRender.^38^ (b) Correlation matrix plot of cell killing, PP, and PpIX fluorescence signals as measured by imaging (fluorescence intensity in pixel value) and spectroscopy analysis. (c) The regression correlation between the increase in PP pixel fluorescence intensity and calcein AM and ethidium bromide signals at $50\;J/{\mathrm{cm}}^{2}$ (n=4) and $100\;J/{\mathrm{cm}}^{2}$ (n=4) PDT treatment.

To correlate imaging readouts with responses in cultures treated with various light doses, alginate scaffolds were dissociated, and cells were collected for viability analysis after treatment (Fig. 6). This procedure was quantitatively analyzed after imaging of vital dye fluorescence as the terminal endpoint. For both viability analyses, the photoproduct accumulation was found to correlate with response to treatment [Figs. 6(b) and 6(c)].

### 3.4 Imaging Photoproduct Accumulation due to ALA-PDT in Murine Xenografts

**Fig. 7:**
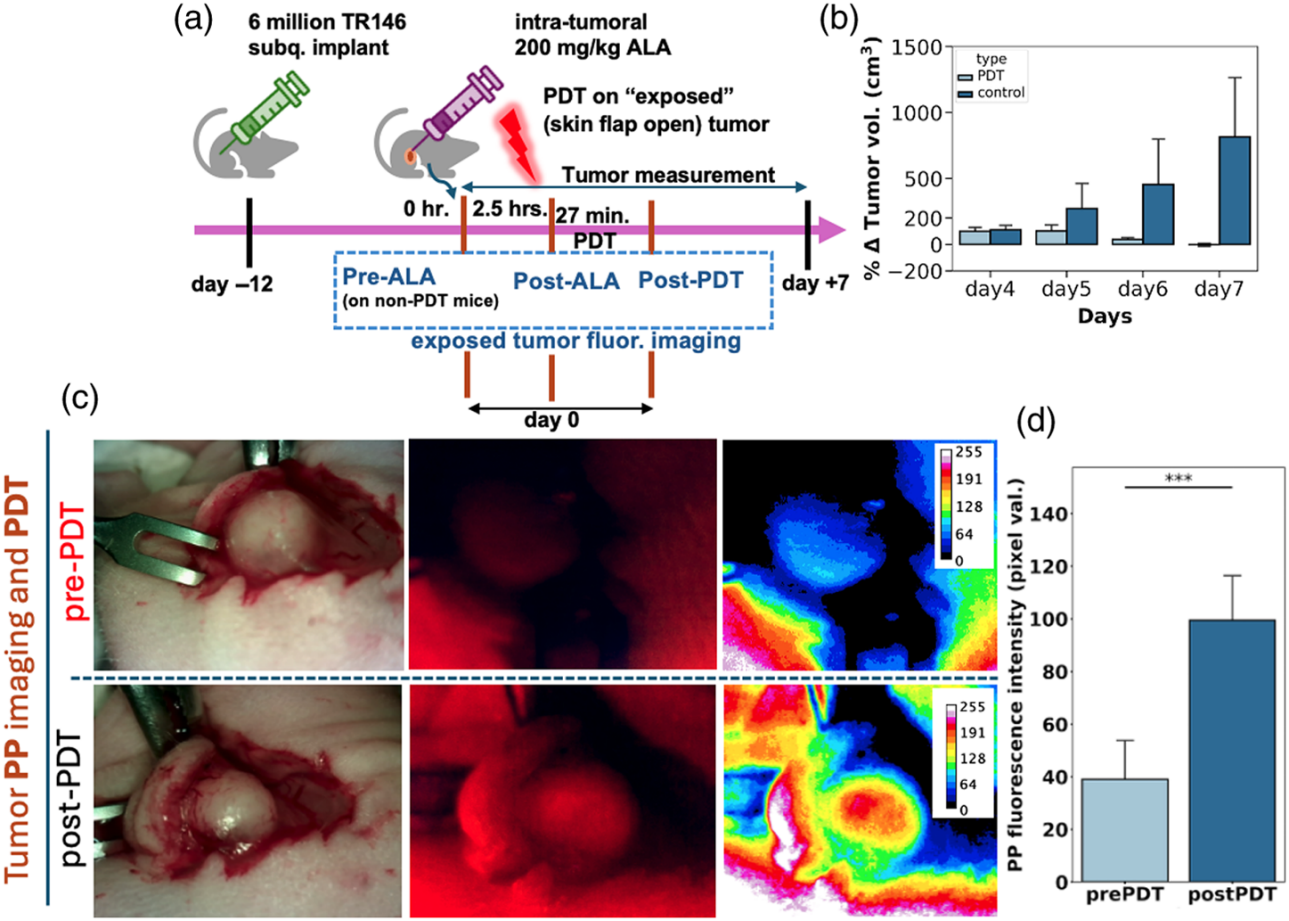
*In vivo* imaging of photoproduct accumulation during PDT. (a) Timeline of tumor implantation, ALA administration, PDT treatment, and PP imaging in TR146 murine xenograft models. (b) Representative polarized white light (pWL), fluorescence, and contrast-enhanced display images of PDT-treated and untreated tumors. (c) Monitoring the progression of tumor volumes after PDT in treated and control animals is based on caliper measurements at each time point (error bars indicate standard deviation). (d) Quantification of average fluorescence photoproduct signal from PDT-treated tumor tissue.

To assess the feasibility of imaging photoproduct as a dosimetry reporter *in vivo*, a small pilot study was conducted using TR146 tumor implants in nude mice. A subcutaneous mouse xenograft skin flap model was adopted and used in the experimental design shown in Fig. 7. For this model, subcutaneous implants were prepared according to a standard protocol.^10^^,^^37^ At the time of PDT, a small incision is made to lift the skin covering the tumor and then later sutured. This procedure allows for fluorescence imaging of the tumor tissue at the time of treatment, whereas the skin flap can be safely sutured after treatment to allow for an extended period of post-treatment monitoring. Although our standard protocol for PDT in subcutaneous xenografts uses transdermal light delivery,^10^^,^^37^ the skin flap is important in the present study to allow imaging of the tumor with 405 and 450 nm excitation wavelengths that would otherwise be highly attenuated by the intact skin over the tumor. Here, a small number of animals were used to establish the feasibility of the protocol, with three animals receiving PDT treatment ($100\;J/{\mathrm{cm}}^{2}$) and two untreated controls (skin flap incision and suturing only).

Images, using the same optical system as for *in vitro* studies, were acquired from the anesthetized mice with skin flap opened immediately prior to ALA injection (for baseline tissue autofluorescence background), again after PpIX accumulation but prior to light delivery, and again immediately following light delivery (Fig. 7). To correlate fluorescence imaging with treatment outcomes, monitoring of tumor volumes commenced after initial inflammation in treated tissue subsided by day 4 post-treatment. Representative white light and PP fluorescence images are shown in Fig. 7(b). As expected for this ALA and PDT dose, the tumor response in treated animals was decisive relative to controls [Fig. 7(c), as well as control mice without ALA induction, see Fig. S8 in the Supplementary Material]. All mice healed well from the skin flap incisions, and there were no complications from suturing, suggesting that this model is suitable for larger-scale *in vivo* assessment of PP as a treatment prognostic.

Further analysis of photobleaching at the tumor site (Fig. S9 in the Supplementary Material) shows the same trend as observed in cell culture models, with treatment resulting in a decreased PpIX signal and an increased signal associated with the PpIX photoproduct. Although it is not possible to establish a dose-dependent correlation with this small number of animals, the clear accumulation of PP in the PDT-treated mice [Fig. 7(d)] is consistent with the trend seen in 3D cell cultures, where PP accumulation is indicative of the light dose deposited.

## 4 Conclusion

Overall, the results of this study suggest that, with further validation data, imaging PpIX photoproduct fluorescence during treatment may be a useful treatment prognostic for image-guided PDT of oral lesions, and potentially any ALA-PDT application for which the appropriate optics can be implemented for PP imaging. In contrast to photobleaching, PP emerges as a distinct optical signal in a different wavelength range. A logical strategy may be to look at the ratio of photoproduct accumulation to PpIX photobleaching, which could create a more decisive signature than either alone. Although an improvised optical system was used here for proof of principle, the capability to measure both signals could be built into the handheld intraoral device, which already houses two cameras. By mounting the appropriate emission filter for each signal on each camera and adding a 450 nm excitation LED to the device’s head, a single compact intraoral probe can be built to monitor multiple signals, serving as the basis for treatment guidance. Alternatively, the PP absorption peak, which emerges at 670 to 675 nm, could be leveraged for deeper tissue fluorescence excitation, potentially used in conjunction with multispectral imaging to resolve the tightly overlapping absorption and emission peaks in the deep red wavelength range. With further *in vivo* validation data, the combined photobleaching/photoproduct optical signal could potentially be used as the basis of a stopping rule to ensure continued light delivery until a curative dose has been deposited. However, one could also extend image-based feedback, if supported by additional correlative datasets, to inform other mid-stream adaptations such as timing of fractionation breaks or adjustment of dose rate to optimize therapy in real time.

## Supplementary Material

10.1117/1.JBO.30.S3.S34114.s01

## Data Availability

All data in support of the findings of this paper are available within the article or as supplementary material.

## References

[1] Bray F. et al., “Global cancer statistics 2022: GLOBOCAN estimates of incidence and mortality worldwide for 36 cancers in 185 countries,” CA Cancer J. Clin. 74(3), 229–263 (2024). CAMCAM 0007-9235 10.3322/caac.21834 38572751

[2] Mello F. W. et al., “Prevalence of oral potentially malignant disorders: a systematic review and meta-analysis,” J. Oral Pathol. Med. 47(7), 633–640 (2018). JPMEEA 0904-2512 10.1111/jop.12726 29738071

[3] Akashanand et al., “Burden of oral cancer and associated risk factors at national and state levels: a systematic analysis from the global burden of disease in India, 1990–2021,” Oral Oncol. 159, 107063 (2024). EJCCER 1368-8375 10.1016/j.oraloncology.2024.107063 39357385

[4] Celli J. P. et al., “Imaging and photodynamic therapy: mechanisms, monitoring, and optimization,” Chem. Rev. 110(5), 2795–2838 (2010). CHREAY 0009-2665 10.1021/cr900300p 20353192 PMC2896821

[5] Grant W. E. et al., “Photodynamic therapy of malignant and premalignant lesions in patients with ’field cancerization‘ of the oral cavity,” J. Laryngol. Otol. 107(12), 1140–1145 (1993). JLOTAX 0022-2151 10.1017/S0022215100125496 8289004

[r6] Siddiqui S. A. et al., “Clinical evaluation of a mobile, low-cost system for fluorescence guided photodynamic therapy of early oral cancer in India,” Photodiagn. Photodyn. Ther. 38, 102843 (2022). 10.1016/j.pdpdt.2022.102843 PMC9177774 35367616

[7] Chen H.-M. et al., “5-Aminolevulinic acid-mediated photodynamic therapy for oral cancers and precancers,” J. Dent. Sci. 7(4), 307–315 (2012). 10.1016/j.jds.2012.03.023

[8] Khan S. et al., “Clinical assessment of a low-cost, hand-held, smartphone-attached intraoral imaging probe for 5-aminolevulinic acid photodynamic therapy monitoring and guidance,” J. Biomed. Opt. 28(8), 082809 (2023). JBOPFO 1083-3668 10.1117/1.JBO.28.8.082809 37483565 PMC10362156

[r9] Khan S. et al., “Clinical evaluation of smartphone-based fluorescence imaging for guidance and monitoring of ALA-PDT treatment of early oral cancer,” J. Biomed. Opt. 25(6), 063813 (2020). JBOPFO 1083-3668 10.1117/1.JBO.25.6.063813 32279466 PMC7148420

[10] Khan S. et al., “Enabling point of care optical diagnostics and treatment of oral lesions in resource-limited settings: preclinical development and evaluation of a low-cost theranostic intraoral device for image-guided photodynamic therapy,” Biophotonics Discov. 2(4), 042305 (2025). 10.1117/1.BIOS.2.4.042305 PMC13038290 41923902

[11] Pogue B. W. et al., “Revisiting photodynamic therapy dosimetry: reductionist &amp; surrogate approaches to facilitate clinical success,” Phys. Med. Biol. 61(7), R57–R89 (2016). PHMBA7 0031-9155 10.1088/0031-9155/61/7/R57 26961864 PMC12153017

[12] Johansson A. et al., “Protoporphyrin IX fluorescence and photobleaching during interstitial photodynamic therapy of malignant gliomas for early treatment prognosis,” Lasers Surg. Med. 45(4), 225–234 (2013). LSMEDI 0196-8092 10.1002/lsm.22126 23533060

[13] Glidden M. D. et al., “Image-based quantification of benzoporphyrin derivative uptake, localization, and photobleaching in 3D tumor models, for optimization of PDT parameters,” Theranostics 2(9), 827–839 (2012). 10.7150/thno.4334 23082096 PMC3475211

[14] Dietel W. Wendenburg R., “Phototransformation of ALA-induced protoporphyrin IX (PPIX) in carcinoma cells and of exogenous PPIX in cells and solutions,” Proc. SPIE 2371, 567–571 (1995). PSISDG 0277-786X 10.1117/12.203418

[r15] Ogbonna S. J. Masuda K. Hazama H., “The effect of fluence rate and wavelength on the formation of protoporphyrin IX photoproducts,” Photochem. Photobiol. Sci. 23(9), 1627–1639 (2024). PPSHCB 1474-905X 10.1007/s43630-024-00611-9 39244727

[r16] Dysart J. S. Patterson M. S., “Photobleaching kinetics, photoproduct formation, and dose estimation during ALA induced PpIX PDT of MLL cells under well oxygenated and hypoxic conditions,” Photochem. Photobiol. Sci. 5(1), 73–81 (2006). PPSHCB 1474-905X 10.1039/b511807g 16395430

[17] Bagdonas S. et al., “Phototransformations of 5-aminolevulinic acid-induced protoporphyrin IX in vitro: a spectroscopic study¶,” Photochem. Photobiol. 72(2), 186–192 (2007). PHCBAP 0031-8655 10.1562/0031-8655(2000)0720186POAAIP2.0.CO2 10946571

[18] Ogbonna S. J. et al., “Increased fluorescence observation intensity during the photodynamic diagnosis of deeply located tumors by fluorescence photoswitching of protoporphyrin IX,” J. Biomed. Opt. 28(5), 055001 (2023). JBOPFO 1083-3668 10.1117/1.JBO.28.5.055001 37197689 PMC10185104

[19] Liu H. et al., “Development and evaluation of a low-cost, portable, LED-based device for PDT treatment of early-stage oral cancer in resource-limited settings,” Lasers Surg. Med. 51(4), 345–351 (2019). LSMEDI 0196-8092 10.1002/lsm.23019 30168618 PMC6934354

[20] Song B. et al., “Mobile-based oral cancer classification for point-of-care screening,” J. Biomed. Opt. 26(6), 065003 (2021). JBOPFO 1083-3668 10.1117/1.JBO.26.6.065003 34164967 PMC8220969

[21] Uthoff R. D. et al., “Point-of-care, smartphone-based, dual-modality, dual-view, oral cancer screening device with neural network classification for low-resource communities,” PLoS One 13(12), e0207493 (2018). POLNCL 1932-6203 10.1371/journal.pone.0207493 30517120 PMC6281283

[22] Pogue B. W. Patterson M. S., “Review of tissue simulating phantoms for optical spectroscopy, imaging and dosimetry,” J. Biomed. Opt. 11(4), 041102 (2006). JBOPFO 1083-3668 10.1117/1.2335429 16965130

[23] Hempstead J. et al., “Low-cost photodynamic therapy devices for global health settings: characterization of battery-powered LED performance and smartphone imaging in 3D tumor models,” Sci. Rep. 5, 10093 (2015). SRCEC3 2045-2322 10.1038/srep10093 25965295 PMC4428052

[24] Bargo P. R. et al., “In vivo determination of optical properties of normal and tumor tissue with white light reflectance and an empirical light transport model during endoscopy,” J. Biomed. Opt. 10(3), 034018 (2005). JBOPFO 1083-3668 10.1117/1.1921907 16229662

[25] Lehtonen S. J. R. et al., “Detection improvement of gliomas in hyperspectral imaging of protoporphyrin IX fluorescence—in vitro comparison of visual identification and machine thresholds,” Cancer Treat. Res. Commun. 32, 100615 (2022). 10.1016/j.ctarc.2022.100615 35905671

[26] Lu H. et al., “Fluorescence spectroscopy study of protoporphyrin IX in optical tissue simulating liquid phantoms,” Materials 13(9), 2105 (2020). MATEG9 1996-1944 10.3390/ma13092105 32370118 PMC7254220

[27] Marois M. et al., “Characterization and standardization of tissue-simulating protoporphyrin IX optical phantoms,” J. Biomed. Opt. 21(3), 035003 (2016). JBOPFO 1083-3668 10.1117/1.JBO.21.3.035003 26968385 PMC5994807

[28] Pogue B. W. et al., “Quantitative hemoglobin tomography with diffuse near-infrared spectroscopy: pilot results in the breast,” Radiology 218(1), 261–266 (2001). RADLAX 0033-8419 10.1148/radiology.218.1.r01ja51261 11152812

[29] Davoudi F. et al., “Alginate-based 3D cancer cell culture for therapeutic response modeling,” STAR Protoc. 2(2), 100391 (2021). 10.1016/j.xpro.2021.100391 33778784 PMC7985559

[r30] Ramdhan T. et al., “Time dependent gelling properties of cuboid alginate gels made by external gelation method: effects of alginate-CaCl2 solution ratios and pH,” Food Hydrocoll. 90, 232–240 (2019). FOHYES 0268-005X 10.1016/j.foodhyd.2018.12.022

[31] Sokolov K. et al., “Realistic three-dimensional epithelial tissue phantoms for biomedical optics,” J. Biomed. Opt. 7(1), 148 (2002). JBOPFO 1083-3668 10.1117/1.1427052 11818022

[32] Liu Y. et al., “Efficacy of photodynamic therapy using 5-aminolevulinic acid-induced photosensitization is enhanced in pancreatic cancer cells with acquired drug resistance,” Photodiagn. Photodyn. Ther. 50, 104362 (2024). 10.1016/j.pdpdt.2024.104362 PMC11645186 39395619

[33] Celli J. P. et al., “An imaging-based platform for high-content, quantitative evaluation of therapeutic response in 3D tumour models,” Sci. Rep. 4, 3751 (2014). SRCEC3 2045-2322 10.1038/srep03751 24435043 PMC3894557

[34] Schindelin J. et al., “Fiji: an open-source platform for biological-image analysis,” Nat. Methods 9(7), 676–682 (2012).1548-7091 10.1038/nmeth.2019 22743772 PMC3855844

[35] Celli J., https://BioRender.com/zzjhoek (2025).

[36] Celli J., https://BioRender.com/e3hk0re (2025).

[37] Mallidi S. et al., “In vivo evaluation of battery-operated light-emitting diode-based photodynamic therapy efficacy using tumor volume and biomarker expression as endpoints,” J. Biomed. Opt. 20(4), 048003 (2015). JBOPFO 1083-3668 10.1117/1.JBO.20.4.048003 25909707 PMC4408448

[38] Celli J., https://BioRender.com/6mtslnr (2025).

