# Supplementary material for: Imaging protoporphyrin IX photoproduct accumulation as a dosimetry reporter for monitoring photodynamic therapy of oral cancer
Source: J Biomed Opt. 2025 Dec 9;30(Suppl 3):S34114. doi: 10.1117/1.JBO.30.S3.S34114 (PMC12688037; doi:10.1117/1.JBO.30.S3.S34114)
Supplement: Supplementary file 1 [file JBO_030_S34114_SD001.pdf]

Supplemental Material:

Imaging protoporphyrin IX photoproduct accumulation as a dosimetry reporter for monitoring photodynamic therapy of oral cancer

Christian Liboy,[a,b**] Shakir Khan,[b,c**] Bofan Song,[d] Deshawn Vega,[b] Mohammad A. Saad,[c] Rongguang Liang,[d] Tayyaba Hasan[c,e] and Jonathan P. Celli[b,c*]

[a]Boston College, Boston, Massachusetts, United States.
[b]University of Massachusetts Boston, Boston, Massachusetts, United States.
[c] Wellman Center for Photomedicine, Massachusetts General Hospital, Harvard Medical School, Boston, Massachusetts, United States.
[d]University of Arizona, Wyant College of Optical Sciences, Tucson, Arizona, United States.
[e]Division of Health Sciences and Technology, Harvard University and Massachusetts Institute of Technology, Cambridge, Massachusetts, United States.

*Corresponding author email: Jonathan.Celli@umb.edu
*** These authors contributed equally*

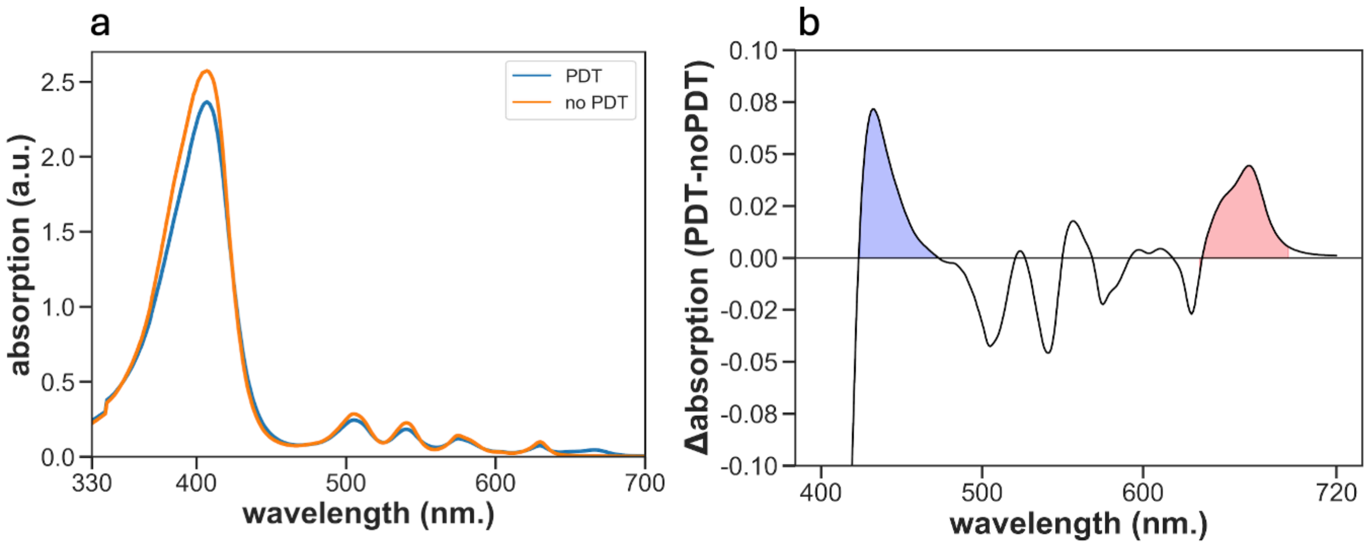

Supplemental Figure 1. The average of four UV-visible absorption spectra (350 nm to 700 nm) for each condition of PpIX in DMSO with and without light exposure (100 J/cm^2). The average data points subtraction spectrum in (b) shows the emergence of new absorption peaks at 450 nm and 673 nm.

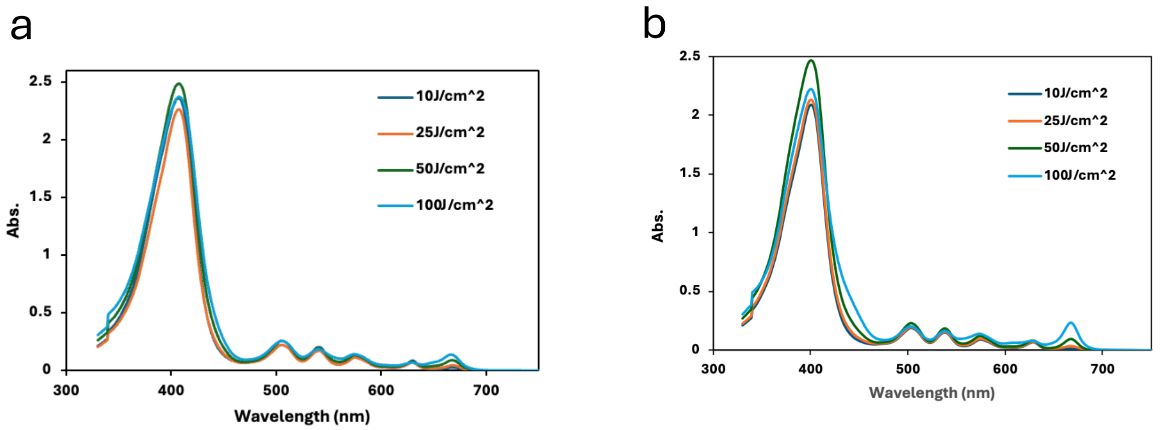

Supplemental Figure 2. Additional UV-Vis spectroscopy data showing fluence-dependent optical properties of PpIX exposed to 635 nm light in (a) DMSO, and (b) Methanol. The new absorption peak associated with photoproduct formation emerges at 673 nm in DMSO, and 667 nm in Methanol.

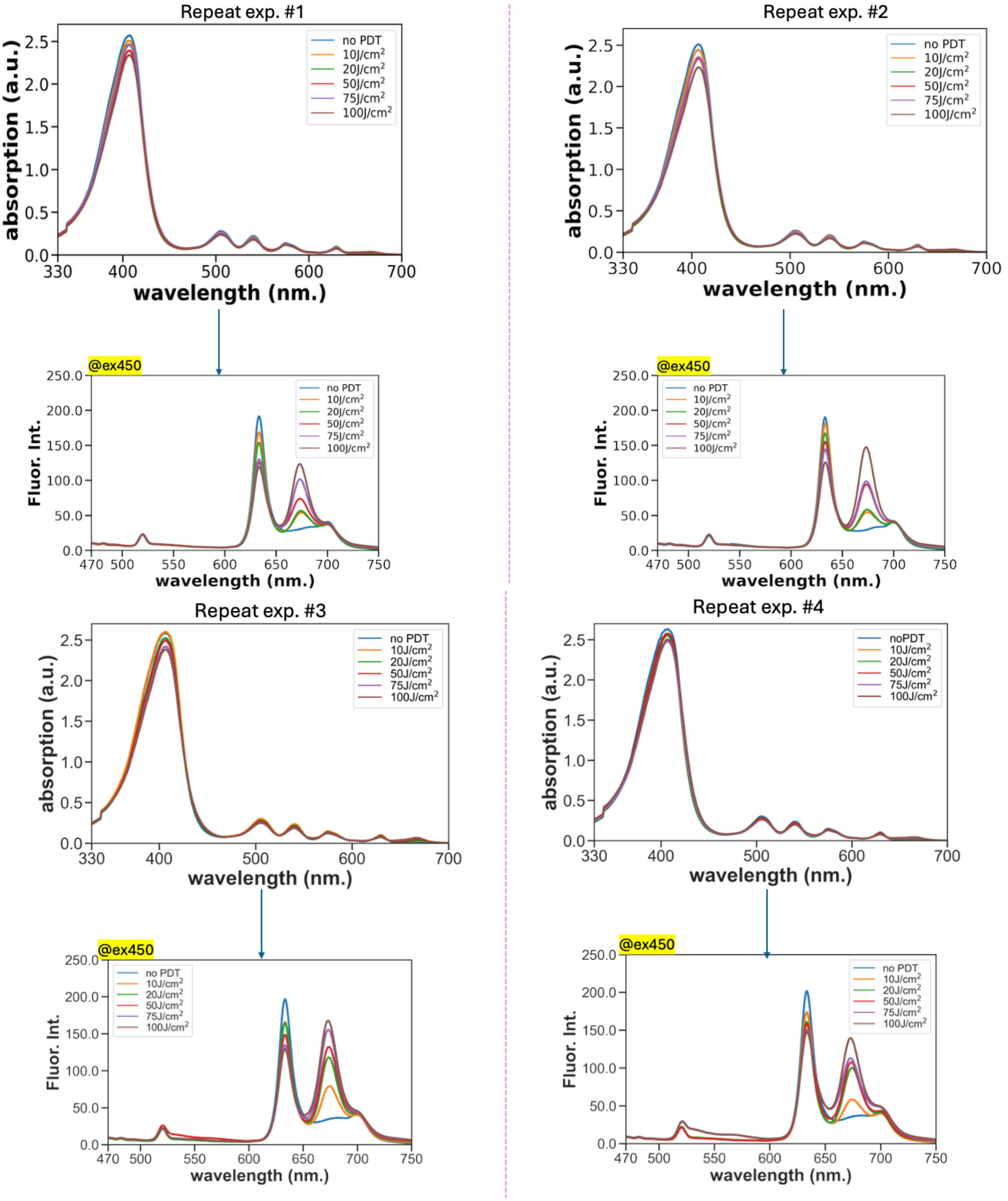

Supplemental Figure 3: *Absorption and fluorescence emission spectra for PpIX in DMSO exposed to varying fluence of 635 nm light. The PDT and spectral reading experiment was repeated 4 times.*

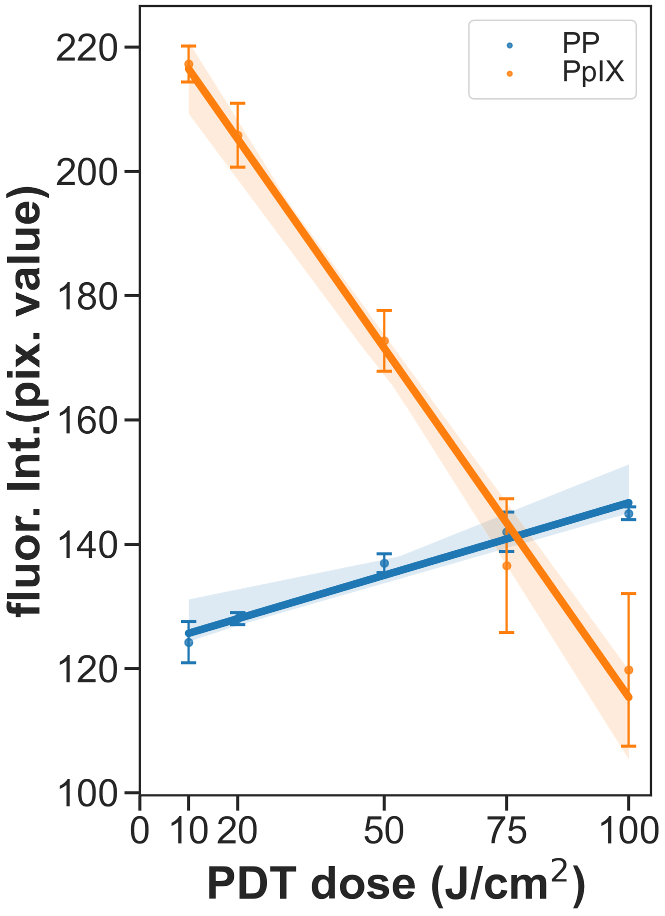

Supplemental Figure 4: Fluence-dependent fluorescence signals for PpIX and PP in solid phantoms (n=3) containing PpIX, hemoglobin and TiO_2 exposed to various doses of 635 nm light.

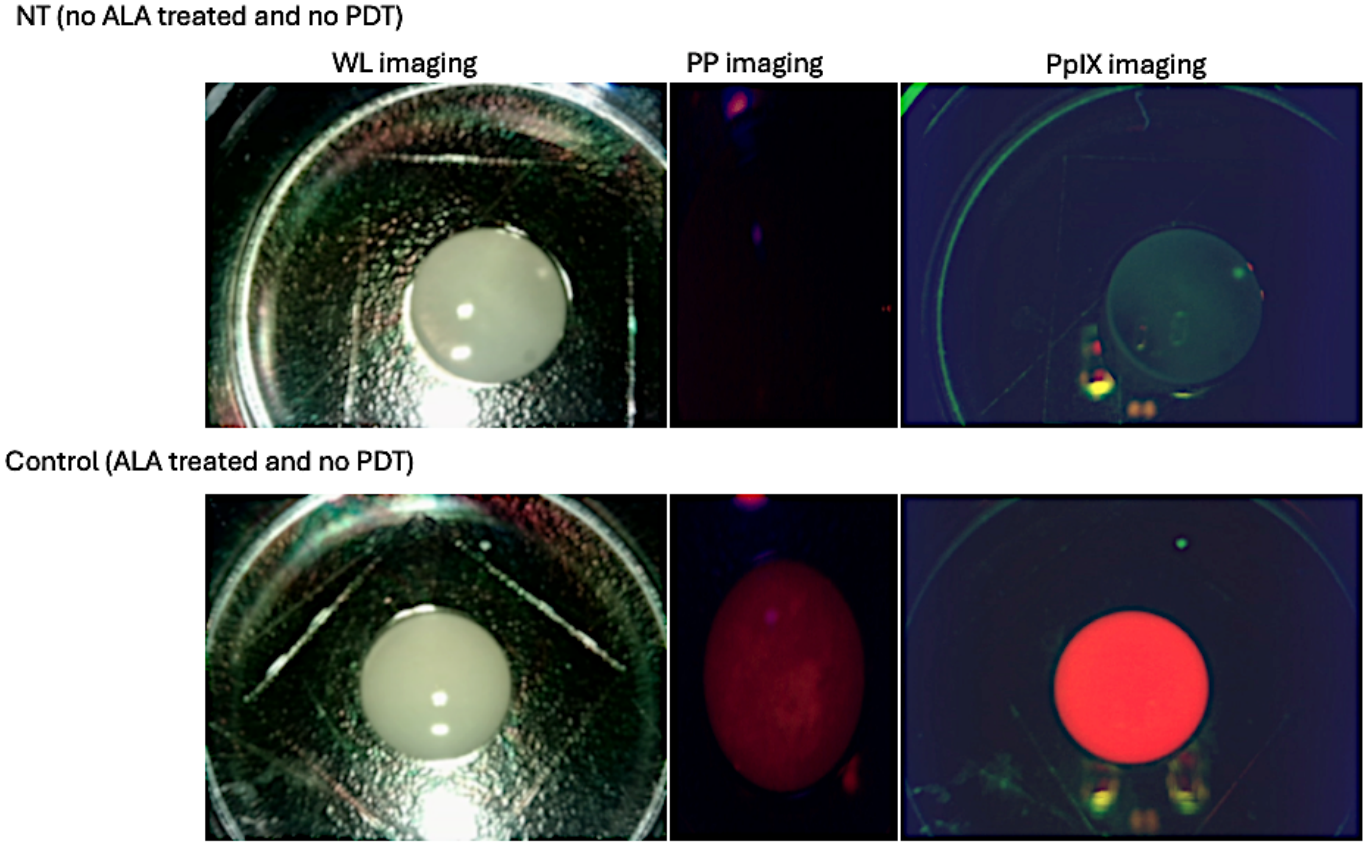

Supplemental Figure 5: Imaging of TR146 OSCC 3D cell cultures using the PpIX and PP imaging optics. Here representative images are shown for cultures without ALA incubation, and with ALA but no light exposure.

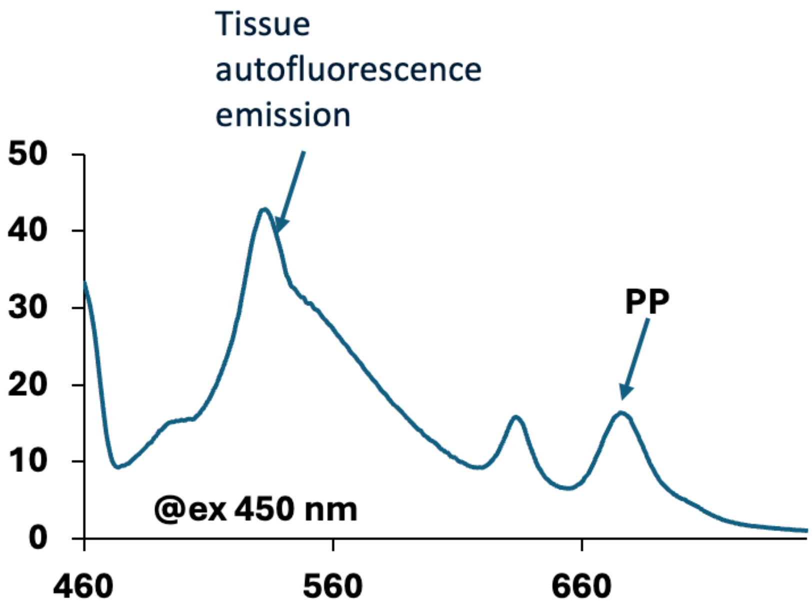

Supplemental Figure 6: Fluorescence emission spectra of ALA-PpIX photosensitized and PDT-treated tissues extracted via lysis. Using 450 nm excitation the PpIX photoproduct peak is clearly visible against autofluorescence signals likely with significant contributions from NADH (reduced nicotinamide adenine dinucleotide) and FAD (flavin adenine dinucleotide) and other endogenous fluorophores.

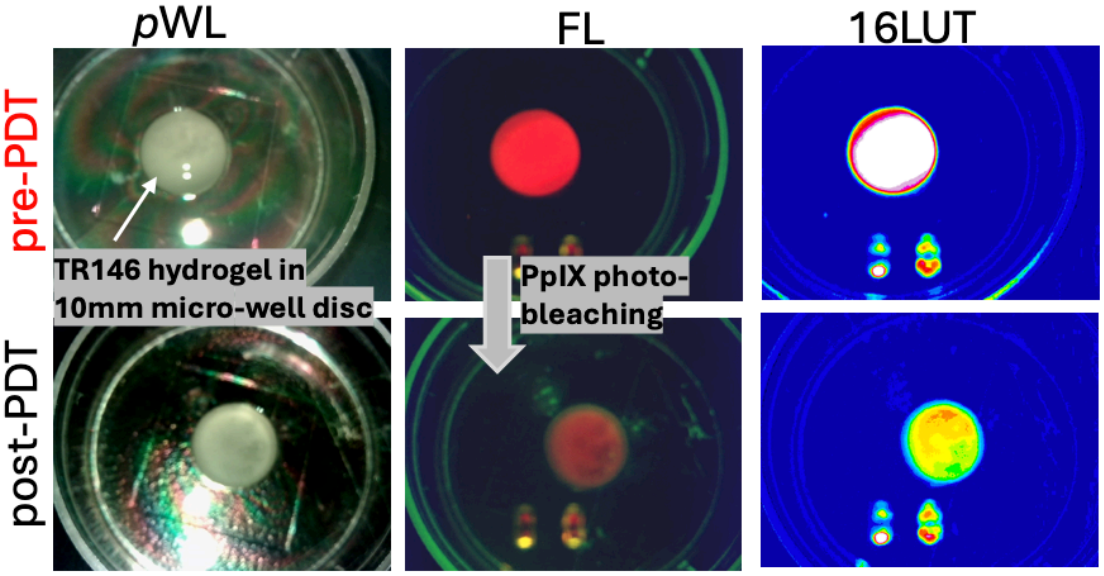

Supplemental Figure 7: Representative images of TR146 3D models obtained using the intraoral camera showing photobleaching of PpIX. The photobleaching here is concomitant with the PP accumulation shown in Figure 4, as expected.

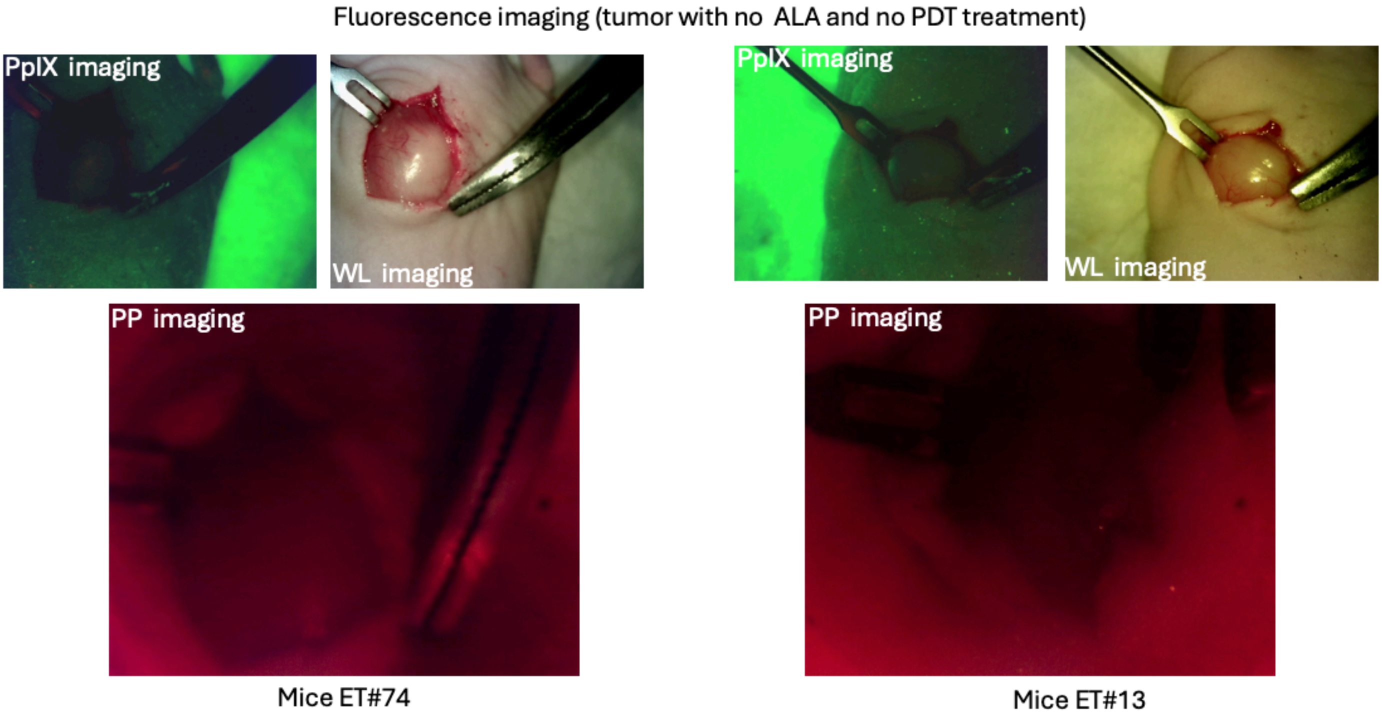

Supplemental Figure 8: Fluorescence imaging of PpIX photoproducts in control mice (no ALA treatment and no light exposure treatment.

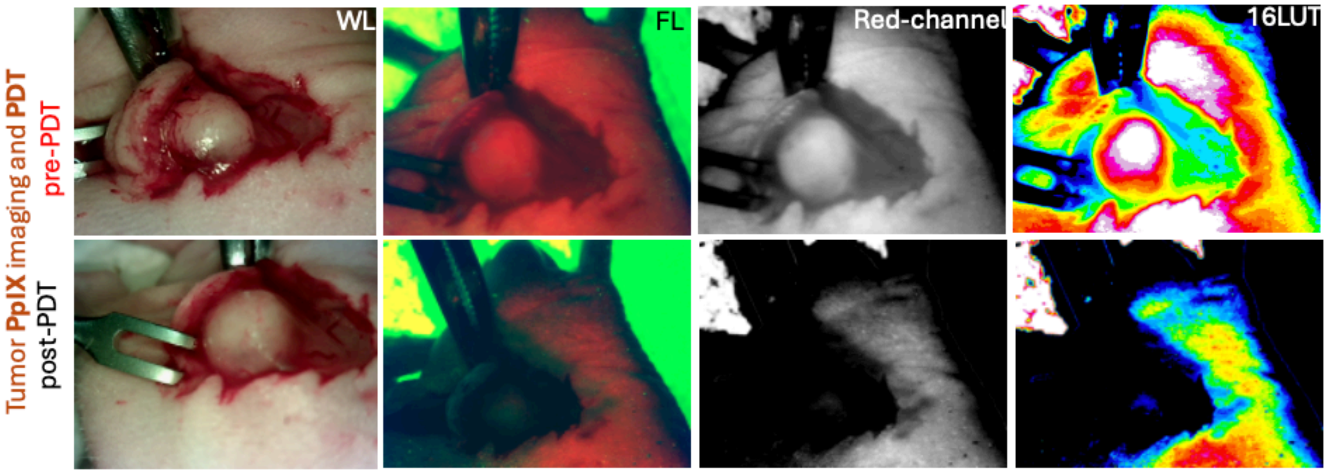

Supplemental Figure 9: Representative images of PpIX photobleaching in murine TR146 xenografts. Images from left to right of the same spatial fields are polarized white light (pWL), multichannel fluorescence (FL) and a contrast-enhanced view of the red channel with 16 color look-up table (16LUT). Photobleaching seen here is consistent with photoproduct formation (Figure 7) in the same spatial fields.
